# Characterization of the changes in supine blood pressure with long‐term use of droxidopa in patients with neurogenic orthostatic hypotension

**DOI:** 10.1002/hsr2.227

**Published:** 2021-01-20

**Authors:** L. Arthur Hewitt, Annika Lindsten, Stephen Gorny, Meghana Karnik‐Henry, Steven Kymes, Antonella Favit

**Affiliations:** ^1^ Lundbeck Deerfield Illinois; ^2^ Lundbeck A/S Copenhagen Denmark

**Keywords:** droxidopa, safety, supine blood pressure, supine hypertension

## Abstract

**Background and Aims:**

Patients with neurogenic orthostatic hypotension (nOH) due to autonomic dysfunction may also experience supine hypertension (defined as supine systolic blood pressure [SBP] ≥140 mmHg). Because pressor agents used to improve nOH symptoms by increasing standing blood pressure (BP) may exacerbate or cause supine hypertension, changes in supine BP with nOH treatments are of interest.

**Methods:**

This post hoc study examined changes in SBP in patients receiving droxidopa (100‐600 mg, three times daily) during a 12‐month long‐term extension study based on whether patients had supine hypertension (ie, supine SBP ≥140 mmHg) at baseline. Shifts from baseline in supine hypertension categorization and mean supine and standing SBP after 6 and 12 months of treatment with droxidopa were determined.

**Results:**

At baseline, 64 patients did not have supine hypertension (mean supine SBP, 120 mmHg) and 38 patients had supine hypertension (mean supine SBP, 157 mmHg). A similar percentage of patients shifted from their respective baseline supine hypertension categorization (ie, with or without supine hypertension) to the other category after receiving droxidopa for 6 or 12 months. After 12 months of droxidopa treatment, patients with supine hypertension at baseline had a mean supine SBP decrease of 3 mmHg and a mean standing SBP increase of 9 mmHg. Patients without supine hypertension at baseline had mean supine and standing SBP increases of 12 and 15 mmHg, respectively.

**Conclusions:**

There was no consistent or progressive elevation in supine SBP over time during the 12‐month treatment with droxidopa in patients either with or without supine hypertension at baseline. These data suggest that long‐term droxidopa treatment for nOH does not adversely affect supine BP.

## 1 INTRODUCTION

Patients with autonomic failure occurring because of neurodegenerative α‐synucleinopathies (eg, Parkinson disease, multiple system atrophy, and pure autonomic failure) often experience neurogenic orthostatic hypotension (nOH), a sustained reduction of blood pressure (BP) on standing.^1^, ^2^, ^3^ The common symptoms of nOH, such as dizziness, lightheadedness, and syncope, can lead to falls and may be associated with impaired function and reduced quality of life.^3^, ^4^ Because of autonomic and baroreceptor dysfunction, patients with nOH can also experience supine hypertension.^1^, ^3^ Although supine hypertension differs from essential hypertension in that the BP elevation in supine hypertension is episodic (ie, not constant as in essential hypertension) and associated with being in the supine position,^1^ supine hypertension may increase the risk of cardiovascular, cerebrovascular, and renal morbidity.^5^, ^6^, ^7^ Supine hypertension and nOH are hemodynamically opposed and, as such, pharmacologic treatments for nOH that increase standing BP may result in or exacerbate supine hypertension.^1^, ^3^, ^8^

Droxidopa is approved by the US Food and Drug Administration to treat symptoms of nOH (dizziness, lightheadedness, or the “feeling that you are about to blackout”) in patients with primary autonomic failure.^9^ The safety and efficacy of droxidopa were examined in two short‐term, phase 3, randomized controlled trials,^10^, ^11^ and patients included in these two trials could continue into a 12‐month, long‐term extension study (NOH303, NCT00738062).^12^ Because droxidopa is a pressor agent that improves symptoms of nOH by increasing standing BP, a greater understanding regarding its effects on changes in supine BP is of interest. Herein, analyses of changes in supine systolic BP (SBP) during 12 months of open‐label treatment with droxidopa in the NOH303 study are reported.

## 2 METHODS

### 2.1 Study design

The design, methods, and results of Study NOH303, an open‐label extension study of treatment with droxidopa in patients with symptomatic nOH, have been previously reported.^12^ Briefly, eligible patients were adults aged ≥18 years with symptomatic nOH associated with primary autonomic failure (Parkinson disease, multiple system atrophy, and pure autonomic failure), dopamine β‐hydroxylase deficiency, or nondiabetic autonomic neuropathy. Patients were included if they showed a symptomatic response (an improvement of ≥1 point in Orthostatic Hypotension Symptom Assessment Item 1 [dizziness/lightheadedness])^13^ to treatment with droxidopa during the 2‐week open‐label titration period in the short‐term randomized trials.^10^, ^11^ Patients received droxidopa at their individual dose (100‐600 mg, three times daily) identified during these short‐term studies.

### 2.2 Analysis of changes in blood pressure

At baseline and at each study visit (which occurred approximately every 4 weeks during the first 3 months of the study, then every 3 months until the end of the study), an orthostatic standing test (OST) was conducted. As part of the OST, BP assessments were taken in the supine position (head and torso elevated 30° from horizontal) at 10 minutes, 5 minutes, and immediately before standing and then 3 minutes after standing. Supine BP is reported as the average of the three OST BP measurements taken in the supine position; standing BP is reported as the OST measurement taken after standing. A mercury, aneroid, or automated digital sphygmomanometer on the arm opposite from which blood samples were drawn was used to measure brachial arterial BP. Throughout the study, the same device was used for each patient, and measurements were always conducted on the same arm.

In this post hoc analysis, patients were categorized according to supine BP at baseline as with or without supine hypertension. Supine hypertension was defined as a supine SBP ≥140 mmHg according to consensus definition.^1^ Changes in supine hypertension categorization (ie, shifts from baseline categorization) and mean supine and standing SBP after 6 and 12 months of treatment with droxidopa were determined. The frequency of SBP measurements >180 mmHg and adverse events (AEs) of hypertension (Medical Dictionary for Regulatory Activities, version 10.1 coding) were also examined.

### 2.3 Ethics

The study protocol was reviewed and approved by an independent ethics committee or institutional review board. The trial was conducted in accordance with local and/or national regulations and principles, the Declaration of Helsinki, and the International Conference on Harmonization Good Clinical Practice Guidelines. Written informed consent was obtained from all patients before enrollment.

## 3 RESULTS

### 3.1 Patients

A total of 102 patients with a mean age of 66 years received ≥1 dose of droxidopa (Table 1). Approximately half of the patients (*n* = 48/102, 47%) had Parkinson disease, and approximately a quarter of the patients (*n* = 27/102, 27%) had multiple system atrophy. The maximum allowed dose of droxidopa (600 mg TID) was used by 38% (*n* = 39/102) of participants. At baseline, the overall mean (SD) supine SBP for the study population was 132.4 (22.4) mmHg (Table 1). The mean (SD) supine SBP at baseline for patients without supine hypertension (*n* = 64) was 118.8 (15.2) mmHg and 155.4 (10.8) mmHg for patients with supine hypertension (*n* = 38). The mean (SD) standing SBPs at baseline in patients without and with supine hypertension were 96.9 (20.0) mmHg and 96.6 (23.7) mmHg, respectively.

**TABLE 1:** Patient demographics and baseline characteristics

Variable	Patients (*n* = 102)
Age, mean (SD), years	65.8 (12.3)
Women, *n* (%)	41 (40.2)
Race, *n* (%)	
White	99 (97.1)
Diagnosis, *n* (%)	
Parkinson disease	48 (47.1)
Multiple system atrophy	27 (26.5)
Pure autonomic failure	18 (17.6)
Other	9 (8.8)
Supine SBP, mean (SD), mmHg	132.4 (22.4)
Baseline SH^a^ groupings	
Without SH^a^	
*n* (%)	64 (62.7)
Supine SBP, mean (SD), mmHg	118.8 (15.2)
Supine SBP, range, mmHg	76.7–138.7
Standing SBP, mean (SD), mmHg^b^	96.9 (20.0)
Standing SBP, range, mmHg^b^	48.0–145.0
With SH^a^	
*n* (%)	38 (37.3)
Supine SBP, mean (SD), mmHg	155.4 (10.8)
Supine SBP, range, mmHg	141.0–184.7
Standing SBP, mean (SD), mmHg^c^	96.6 (23.7)
Standing SBP, range, mmHg^c^	58.0–148.0

Abbreviations: SBP, systolic blood pressure; SH, supine hypertension.

^a^ SH defined as a supine SBP ≥140 mmHg.^1^

^b^ *n* = 63.

^c^ *n* = 36.

At 6 and 12 months, 67 (66%) and 57 (56%) patients remained in the study, respectively. The most common reasons for study discontinuation were AEs (*n* = 20, with no individual AE leading to discontinuation in >1 patient), withdrawal of consent (*n* = 16), and treatment failure/lack of efficacy (*n* = 7).

### 3.2 Changes in systolic blood pressure

**FIGURE 1:**
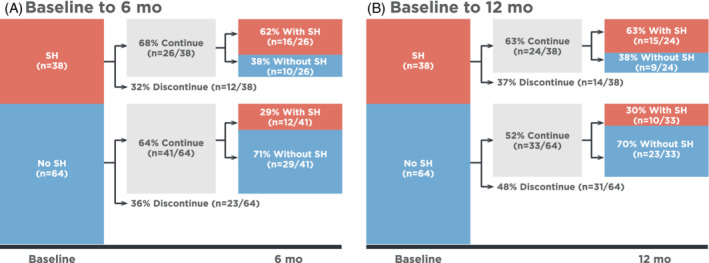
Changes in SH (SH defined as an SBP ≥140 mmHg in the supine position^1^) classification from baseline to, A, 6 and B, 12 months. SBP, systolic blood pressure; SH, supine hypertension

Of patients without supine hypertension at baseline, approximately one‐third transitioned to supine hypertension after 6 and 12 months of treatment with droxidopa (Figure 1A,B). Of patients with supine hypertension at baseline, 38% transitioned to not having supine hypertension after 6 and 12 months of treatment with droxidopa (Figure 1A,B).

**FIGURE 2:**
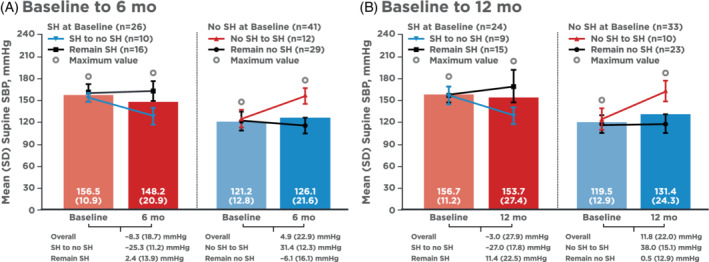
Mean (SD) changes in supine SBP by SH (SH defined as an SBP ≥140 mmHg in the supine position^1^) classification from baseline to, A, 6 and B, 12 months. Bars indicate mean (SD) for the entire baseline group. Connected lines indicate mean (SD) change by classification subgroup. SBP, systolic blood pressure; SH, supine hypertension

Patients with supine hypertension at baseline had mean (SD) supine SBP reductions of −8.3 (18.7) and −3.0 (27.9) mmHg after 6 and 12 months of treatment with droxidopa, respectively (Figure 2A,B). Patients without supine hypertension at baseline had mean (SD) supine SBP increases of 4.9 (22.9) and 11.8 (22.0) mmHg after 6 and 12 months of treatment with droxidopa, respectively (Figure 2A,B).

Patients with supine hypertension at baseline had mean (SD) standing SBP increases of 8.3 (25.6) and 9.2 (30.6) mmHg after 6 and 12 months of treatment with droxidopa, respectively (Table 2). In patients without supine hypertension at baseline, mean (SD) standing SBP increases were 11.8 (25.5) and 14.6 (23.5) mmHg.

**TABLE 2:** Changes from baseline in standing SBP^a^

	Patients without SH^b^	Patients with SH^b^
6 months		
*n*	41	26
Mean (SD) change in standing SBP, mmHg	11.8 (25.5)	8.3 (25.6)
12 months		
*n*	33	24
Mean (SD) change in standing SBP, mmHg	14.6 (23.5)	9.2 (30.6)

Abbreviations: SBP, systolic blood pressure; SH, supine hypertension.

^a^ After 3 minutes during orthostatic standing test.

^b^ SH defined as a supine SBP ≥140 mmHg.^1^

### 3.3 Safety related to BP elevations

**FIGURE 3:**
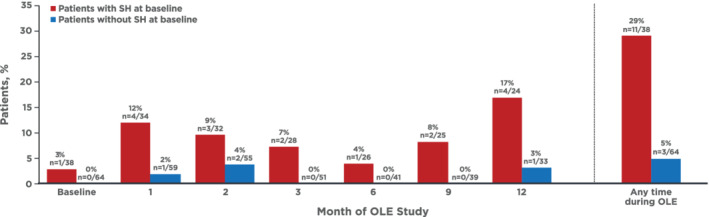
Supine SBP measurements >180 mmHg by baseline SH (SH defined as an SBP ≥140 mmHg in the supine position^1^) classification. OLE, open‐label extension; SBP, systolic blood pressure; SH, supine hypertension

Throughout the study, supine BP measurements >180 mmHg were more commonly observed in patients with supine hypertension at baseline (3%–17%) compared with patients without supine hypertension (0%–4%; Figure 3). Cumulatively, supine BP measurements >180 mmHg were observed in 14% (*n* = 14/102) of patients at any time during the study (5% [*n* = 3/64] in patients without supine hypertension at baseline; 29% [*n* = 11/38] in patients with supine hypertension at baseline; Figure 3).

The frequency of AEs related to elevations in BP was low and considered mild or moderate in severity. Hypertension was reported as an AE by two patients and led to the withdrawal of one of these patients from the study. The hypertension AE leading to withdrawal was considered to be of moderate intensity and possibly related to droxidopa by the investigator.

## 4 DISCUSSION

In this 12‐month study of treatment with droxidopa in patients with nOH, limited changes from baseline in supine SBP were observed. Although patients with and without supine hypertension had standing SBP increases from baseline after 6 and 12 months of droxidopa, there were disparate effects on supine BP. Patients without supine hypertension at baseline experienced increased supine SBP during treatment with droxidopa, likely because of its pressor activity, but the elevations were not consistent or clinically significant and potentially represent patient‐specific random incidence. In contrast, mean decreases in supine SBP during treatment with droxidopa were observed for the group with supine hypertension at baseline even though the group had increases in standing SBP (8–9 mmHg) generally consistent with those previously identified with droxidopa treatment (12 mmHg in the pooled clinical trial population^14^). These findings are interesting and are an area of potential investigation to determine whether they are related to therapeutic effects (eg, improved overall BP regulation due to treatment of nOH) or represent a statistical phenomenon (eg, regression to the mean). These results underscore the importance of BP monitoring in patients treated with droxidopa to achieve a balance between nOH symptom improvement and supine hypertension risk minimization to avoid potential negative clinical outcomes associated with each condition.^4^, ^5^, ^8^

During the 12 months of treatment with droxidopa in this study, only two patients reported an AE of hypertension, of which one event was moderate in severity, possibly related to droxidopa, and resulted in study discontinuation. The AEs in this study have been described in detail previously.^12^ The most commonly reported AEs were falls, urinary tract infections, headache, syncope, and back pain.^12^ Most AEs were considered unrelated or unlikely to be related to treatment. Many of the most commonly reported AEs are not unexpected in the patient population studied because of older age and underlying neurodegenerative disease.

The changes in supine BP and safety in patients treated with droxidopa observed in the current analyses are consistent with previously reported outcomes. Another long‐term study of droxidopa reported that 5% of patients (*n* = 17/350) experienced AEs of hypertension (21 total events) and four patients discontinued the study because of hypertension‐related reasons.^15^ Although pooling the BP data from that study with the data from the current study might be of interest, these analyses are not possible because of differences in BP collection methods between the studies (eg, collection time points and body position).

Although the results of the current analysis provide further information on the effects of droxidopa on supine BP, some limitations warrant cautious interpretation. These include the relatively small sample sizes examined and the post hoc nature of the analyses. Additionally, our findings may be limited by the number of patients who discontinued before the end of the study (*n* = 45/102, 44%). However, this discontinuation rate is not unexpected for a study with a relatively long (12‐month) duration in a patient population that is older and medically complex and with underlying neurodegenerative diseases with autonomic failure. Further, discontinuation data suggest no fundamental concerns about the long‐term efficacy or tolerability of droxidopa. During the 12‐month study period, relatively few patients discontinued for reasons attributed to inadequate therapeutic effects (*n* = 7/102), and no individual AE led to discontinuation of >1 patient in those who discontinued owing to AEs (*n* = 20/102).

In conclusion, patients with nOH may have varied responses to droxidopa that could, in part, be related to their baseline supine SBP. No large BP fluctuations were observed, and importantly, there was no evidence of a consistent or progressive elevation in supine BP over time. BP‐related AEs were infrequent and typically reported to be mild to moderate in severity. Taken together, these data suggest that long‐term treatment with droxidopa for nOH does not adversely affect supine BP.

## FUNDING

The data reported in this manuscript were derived from a research project funded by Lundbeck.

## CONFLICT OF INTEREST

All authors are employees of Lundbeck.

## AUTHOR CONTRIBUTIONS

Conceptualization: L. Arthur Hewitt, Annika Lindsten, Stephen Gorny, Meghana Karnik‐Henry, Steven Kymes, Antonella Favit

Data curation: Annika Lindsten, Meghana Karnik‐Henry

Formal analysis: Annika Lindsten

Investigation: L. Arthur Hewitt, Stephen Gorny

Methodology: L. Arthur Hewitt, Annika Lindsten, Steven Kymes

Project administration: L. Arthur Hewitt, Stephen Gorny

Software: Annika Lindsten

Supervision: L. Arthur Hewitt, Stephen Gorny

Validation: Annika Lindsten, Steven Kymes

Writing ‐ original draft preparation: Annika Lindsten, Meghana Karnik‐Henry

Writing ‐ review and editing: L. Arthur Hewitt, Annika Lindsten, Stephen Gorny, Meghana Karnik‐Henry, Antonella Favit, Steven Kymes

  All authors have read and approved the final version of the manuscript.

  L. Arthur Hewitt had full access to all of the data in this study and takes complete responsibility for the integrity of the data and the accuracy of the data analysis.

## TRANSPARENCY STATEMENT

L. Arthur Hewitt, the corresponding author, affirms that this manuscript is an honest, accurate, and transparent account of the study being reported; that no important aspects of the study have been omitted; that any discrepancies from the study as planned have been explained.

## Data Availability

The data that support the findings of this study are available from the corresponding author upon reasonable request.
